# Optimal Dietary Replacement of Fishmeal With *Spirulina platensis* Enhances Growth Performance, Digestion, Antioxidant Capacity, Ovarian Development, and Muscle Quality in *Macrobrachium nipponense*

**DOI:** 10.1155/anu/7865897

**Published:** 2026-06-28

**Authors:** Longyu Liu, Anran Zou, Weihong Zhao, Tianqi Cao, Fengjuan Jiang, Da Yue, Ling Yin, Wenjing Wu, Chuanjie Qin, Zhigang Gao

**Affiliations:** ^1^ College of Marine and Bioengineering, Yancheng Institute of Technology, Yancheng, 224051, China, ycit.edu.cn; ^2^ School of Marine Science and Environment Engineering, Dalian Ocean University, Dalian, 116023, China, dlou.edu.cn; ^3^ Dongtai Cibainian Bioengineering Co., Ltd., Yancheng, 224051, China; ^4^ College of Applied Chemistry and Environmental Engineering, Yancheng Teachers University, Yancheng, 224051, China, yctc.edu.cn; ^5^ Key Laboratory of Sichuan Province for Fishes Conservation and Utilization in the Upper Reaches of the Yangtze River, Neijiang Normal University, Neijiang, 641100, China, njtc.edu.cn

**Keywords:** activities of SOD and CAT, digestive enzyme, fishmeal replacement, growth performance, *Macrobrachium nipponense*, muscle quality, ovarian development, *Spirulina platensis*

## Abstract

This study evaluated the optimal replacement level of fishmeal with *Spirulina platensis* in diets for *Macrobrachium nipponense*, focusing on its comprehensive effects on growth performance, hepatopancreatic catalase (CAT) and superoxide dismutase (SOD) activities, ovarian development, and muscle amino acid composition. Six isonitrogenous and isolipidic diets were formulated, replacing 0%, 20%, 40%, 60%, 80%, and 100% of the fishmeal with *S. platensis*. A 30‐day feeding trial, which spanned the critical period of ovarian development, was conducted to evaluate these diets. Results showed that substitution levels up to 60% maintained growth and survival comparable to the control group, while levels of 80% and 100% resulted in poor survival and growth. The 60% replacement group demonstrated the highest activities of key digestive enzymes (trypsin, lipase, and amylase) and the peak activities of SOD and CAT. Diets containing spirulina exhibited a significantly higher ovarian maturation rate than the control (*p* < 0.05). Muscle amino acid analysis revealed that high substitution levels (≥60%) decreased arginine but increased proline content, with the 100% group showing significant reductions in total essential amino acids (EAAs), arginine, methionine, threonine, leucine, and lysine (*p* < 0.05). This study demonstrates that substituting up to 60% of fishmeal with *S. platensis* is a viable strategy for *M. nipponense* aquaculture, which delivers comprehensive benefits, including supported growth, enhanced health, and promoted ovarian development.

## 1. Introduction

Fishmeal is regarded as an ideal protein source in aquaculture, given its balanced profile of essential amino acids (EAAs), fatty acids, minerals, and bioactive compounds, coupled with low levels of antinutritional factors [1, 2]. Aquaculture now accounts for 86% of its global consumption [3], driving annual demand to an estimated 3.9 million metric tons [4, 5]. This growing reliance, however, is fundamentally challenged by climate change and fishing restrictions that are diminishing the supply of raw materials, thereby rendering the development of alternative protein sources an urgent priority [6].

*Spirulina platensis* presents a compelling nutritional profile for aquaculture, not only matching fishmeal in core components—such as its 60%–70% protein content and abundance of vitamins, minerals, and essential fatty acids but also supplementing it with unique bioactive compounds, including antioxidant pigments and carotenoids [5, 7]. Beyond composition, its protein exhibits higher digestibility due to soft peptidoglycan cell walls, in contrast to the rigid cellulose‐based walls of other microalgae, which enhances nutrient assimilation in most fish species [8]. The commercial viability of spirulina is further strengthened by its rapidly expanding production, which increased from 13,156 tonnes in 2018 to 18,089 tonnes (dry weight) in 2024 [5, 9, 10]. These attributes are substantiated by a growing body of research demonstrating that dietary inclusion of *S. platensis* improves growth performance, immune response, and muscle quality across diverse aquatic species [11–16], collectively positioning it as a highly viable and multifunctional alternative to fishmeal. Moreover, spirulina has been shown to enhance reproductive performance, including gonadal development, fecundity, and spawning performance [13, 17, 18].

The oriental river prawn (*Macrobrachium nipponense*), an economically significant species in Asia, however, exhibits a precocious maturation problem: a considerable proportion of females mature at undersized dimensions, accompanied by underdeveloped ovaries and inefficient spawning, which collectively constrain consistent larval production and sustainable stock replenishment [19, 20]. In commercial practice, farmers and breeders seek to achieve both optimal ovarian development and maximum body growth. Precocious maturation is influenced by multiple factors, with nutritional quality—particularly dietary protein sources and functional nutrients‐recognized as a key determinant [21–23]. Therefore, when investigating the replacement of fishmeal with spirulina, attention must be paid to both growth performance and ovarian development. Only under the precondition that growth requirements are adequately met can the promotion of ovarian development be regarded as meaningful. It is therefore of interest to investigate whether dietary inclusion of spirulina as a fishmeal alternative affects ovarian development in *M. nipponense*, whether such effects are beneficial when growth performance is maintained.

To comprehensively assess its applicability in oriental river prawn aquaculture, this study systematically evaluates the effects of replacing fishmeal with *S. platensis* in *M. nipponense* diets, with specific emphasis on (1) growth performance, (2) digestive capacity and activities of superoxide dismutase (SOD) and catalase (CAT), (3) ovarian development, and (4) muscle nutritional quality (with a focus on amino acid composition). The findings are expected to provide critical insights for formulating sustainable feeding strategies that concurrently address fishmeal replacement needs and reproductive management challenges, thereby contributing to improved health and productivity in farmed prawns.

## 2. Materials and Methods

### 2.1. Experimental Diets

The composition of the experimental diets is detailed in Table 1, and the formulation was based on established references [25]. Fishmeal, soybean meal, and brewer’s yeast were employed as dietary protein sources. Six isonitrogenous and isolipidic diets were meticulously crafted with varying levels of *S. platensis* replacing fishmeal protein 0% (SP0, control group), 20% (SP20), 40% (SP40), 60% (SP60), 80% (SP80), and 100% (SP100).

**Table 1:** Ingredient and chemical composition of the experimental diets containing different levels of dietary *S. platensis*.

Ingredients (%)	Diets					
	SP0	SP20	SP40	SP60	SP80	SP100
*S. platensis*	0.00	8.40	16.80	25.20	33.80	42.07
Fish meal	40.00	32.00	24.00	16.00	8.00	0.00
Soybean meal	8.00	8.00	8.00	8.00	8.00	8.00
Rapeseed meal	8.00	8.00	8.00	8.00	8.00	8.00
Beer yeast	12.00	12.00	12.00	12.00	12.00	12.00
α‐Cellulose	17.00	17.00	17.00	17.00	17.00	17.00
Cholesterol	0.50	0.50	0.50	0.50	0.50	0.50
Monocalcium phosphate	1.30	1.30	1.30	1.30	1.30	1.30
Salt	0.30	0.30	0.30	0.30	0.30	0.30
Betaine	1.00	1.00	1.00	1.00	1.00	1.00
Vitamins and minerals	4.00	4.00	4.00	4.00	4.00	4.00
Fish oil	2.40	3.10	3.80	4.45	5.15	5.83
Cellulose	5.50	4.40	3.30	2.25	0.95	0.00
Composition (%)^a^						
Crude protein	39.68	39.23	40.29	40.10	39.76	40.54
Crude lipid	6.24	6.37	6.20	5.89	5.78	6.02
Moisture	7.82	7.70	7.41	8.02	8.24	8.29
Ash	12.56	12.49	12.22	11.95	11.66	11.30
Histidine	0.99	0.95	0.90	0.85	0.81	0.76
Arginine	2.28	2.23	2.18	2.13	2.08	2.03
Threonine	1.48	1.53	1.58	1.63	1.68	1.72
Valine	1.83	1.85	1.87	1.89	1.92	1.94
Methionine	0.82	0.78	0.74	0.70	0.66	0.62
Phenylalanine	1.33	1.38	1.43	1.48	1.54	1.59
Isoleucine	1.51	1.52	1.55	1.58	1.61	1.63
Leucine	2.33	2.37	2.44	2.50	2.55	2.62
Lysine	2.40	2.31	2.18	2.07	1.97	1.83
Tyrosine	1.06	1.12	1.17	1.22	1.27	1.32
Cystine	0.38	0.39	0.40	0.40	0.41	0.42

*Note:* Vitamin mixture supplied the following (mg/g of mixture): thiamin, 5.86; riboflavin, 12.77; nicotinic acid, 67.74; pantothenic acid, 28.14; pyridoxine, 9.38; folic acid, 1.56; cyanocobalamin, 7.66; retinol acetate, 6.13; cholecalciferol, 3.06; hemodal, 8.92; biotin, 3.83; and inose, 78.97; all ingredients were diluted with α‐cellulose to 1 g; mineral mixture supplied the following (mg/g of mixture): NaH_2_PO_4_, 100; KH_2_PO_4_, 215; Ca(H_2_PO_4_)_2_·H_2_O, 265; CaCO_3_, 105; KCl, 28; MgSO_4_·7H_2_O, 100; AlCl_3_·6H_2_O, 12; ZnSO_4_ · 7H_2_O, 5.11; MnSO_4_·6H_2_O, 1.43; KI, 0.58; CaCl_2_, 0.51; CoCl_2_·6H_2_O, 1.76; Ca(CH_3_CHOHCOO)_2_·5H_2_O, 165; and FeC_6_H_5_O·nH_2_O, 0.61 [24].

^a^All dietary composition values presented are measured values.

Each diet’s ingredients were first ground using a 100‐mesh sieve. Phosphoric acid, calcium dihydrogen phosphate, premixed vitamins and minerals, betaine, and microcrystalline cellulose were then uniformly mixed according to the formula. Subsequently, the major ingredients were incrementally added and mixed thoroughly. After mixing, an appropriate amount of water and fish oil was added, and the mixture was kneaded. The resulting mixture was processed into 1 mm diameter pellets using a feed pelletizer. These pellets were then subjected to 24 h in a forced‐air oven maintained at 55°C and subsequently stored in hermetic plastic bags within a freezer at a temperature of 4°C until required.

### 2.2. Prawn and Experimental Conditions

Healthy prawns (0.74 ± 0.08 g) used in the experiment were obtained from the Dazhong Lake, Yancheng City, Jiangsu Province, China. Before the experiment, the prawns were acclimated for 1 week in seven tanks (97.0 cm × 75.5 cm × 66.0 cm) under laboratory conditions and fed the SP0 diet. All tanks were equipped with automatic aerators to maintain dissolved oxygen levels above 6.5 mg/L, a 12 h light/12 h dark photoperiod, and a water temperature of 25 ± 1°C. Following acclimation, 540 female prawns at stage Ⅱ of ovarian development were randomly assigned to six groups with triplicate replicates per group (90 prawns/group) in 18 glass tanks (68 cm × 47 cm × 30 cm), with 1/3 water changed daily by siphoning. Each tank was equipped with four PVC pipes (3 cm in diameter and 6 cm in length) as shelters to prevent the prawns from cannibalizing each other. The prawns were fed 3% of their body weight twice daily (2/3 at 9:00 a.m. and 1/3 at 5:00 p.m.). The environmental conditions were the same as those during the acclimation period. The 30‐day feeding trial was specifically designed to cover the critical period of ovarian development in *M. nipponense* as its ovarian cycle from stage II to maturation typically completes within this timeframe [26, 27]. A 30‐day period has been successfully used in reproductive physiology studies targeting gonadal development in *M. nipponense* [28], and longer trials may introduce confounding effects from multiple molting cycles and natural senescence. Unused feed, feces, and deceased prawns were removed by siphoning approximately 3 h postfeeding. At the end of the experiment, the prawns were fasted for 24 h, sterilized with 75% ethanol, and dissected. Hepatopancreas samples were collected from seven prawns per tank, pooled, and stored at −80°C for subsequent analysis of amylase, lipase, trypsin, SOD, and CAT activities. Each group consisted of three biological replicates, with each assay performed in duplicate. Additionally, muscle samples from the abdominal region were taken from seven prawns per tank for amino acid content analysis, with three biological replicates per group.

### 2.3. Growth Performance

The final weight and number of prawns in each tank were determined. Weight gain and survival rate were calculated as follows:

Survival rate %=100×Final prawn number/initial prawn number,

Weight gain rate %=100×Final weightg -  initial weightg/ initial weightg,

SGR %/day=lnfinal body weight - lninitial body weight/days×100.

### 2.4. Digestive Enzyme Analyses

The hepatopancreas tissue was weighed and homogenized in 10 volumes of saline using a tissue homogenizer (German IKAT10) on ice and centrifuged at 2500 r/min at 4°C for 10 min. The resulting supernatant was collected for subsequent determination of digestive enzyme activities. The activities of digestive enzymes, including amylase, lipase, and trypsin, were detected based on colorimetric methods with kits according to the manufacturer’s instructions (Nanjing Jiancheng Bioengineering Institute, China). Total protease activity was determined by the casein hydrolysis method described by Furné et al. [29] using a total protein kit (Nanjing Jiancheng Bioengineering Institute, China). All digestive enzyme activities were reported in units per milligram of protein.

### 2.5. Hepatopancreatic CAT and SOD Activities

The preparation of the hepatopancreas homogenate was carried out as described in section 2.4. The activities of CAT and SOD were assessed using kits (Nanjing Jiancheng Bioengineering Institute, China), following the manufacturer’s instructions. CAT activity was measured by following hydrogen peroxide reduction at 405 nm according to Aebi [30] and expressed as a unit per milligram of protein. SOD activity was determined by its capability to inhibit the superoxide anion generated within a xanthine and xanthine oxidase reaction system at 550 nm, following the protocol established by McCord and Fridovich [31]. SOD activity was also reported as a unit per milligram of the protein.

### 2.6. Ovarian Development

Ovarian development was assessed at the end of the trial via a noninvasive visual method, which is feasible due to the transparent cephalothorax of *M. nipponense* and is a well‐established practice for this species [32, 33]. The developmental stage was determined by examining the ovary’s color and morphology through the exoskeleton based on distinct color changes Stage I (colorless transparency), II (white), III (khaki), IV (green), and V (dark green, filling the entire cephalothoracic cavity), according to established criteria [26, 27, 34]. To validate the visual staging method, the gonadosomatic index (GSI) was calculated for five representative individuals from each developmental stage (Stages I–V). The GSI was calculated as follows:

GSI %=Ovary weight/body weight×100.

Ovarian maturation was quantified using the following metrics:

Ovarian maturation rate %=Number of females with ovaries at stage V/ Total number of survival females×100.

### 2.7. Amino Acid Analysis

Sampled muscle tissues were dried in an oven at 60°C for 48 h. The samples were digested with 6M aqueous hydrochloric acid and dried under vacuum. The powdered samples obtained were collected for amino acid profile analysis. The amino acid level was determined using an automatic amino acid analyzer (Hitachi Model 835‐50; Japan) equipped with a column for physiological fluid analysis (2.6–150 mm, Hitachi custom ion‐exchange resin no. 2619 Tokyo, Japan). Samples were hydrolyzed in 6N HCl at 110°C for 22 h, then separated by the ion‐exchange column, reacted with ninhydrin solution, and the amino acid levels were quantified through spectrophotometry.

### 2.8. Statistical Analysis

The results were presented as mean ± standard error (SE) and were examined for homogeneity of variances (F‐test). To compare the effects of dietary treatments on all parameters measured, data were subjected to one‐way analysis of variance (ANOVA) followed by Duncan’s test. A significance level of *p*  < 0.05 was used for all statistical tests.

## 3. Results

### 3.1. Growth and Survival Performance

**Figure 1:**
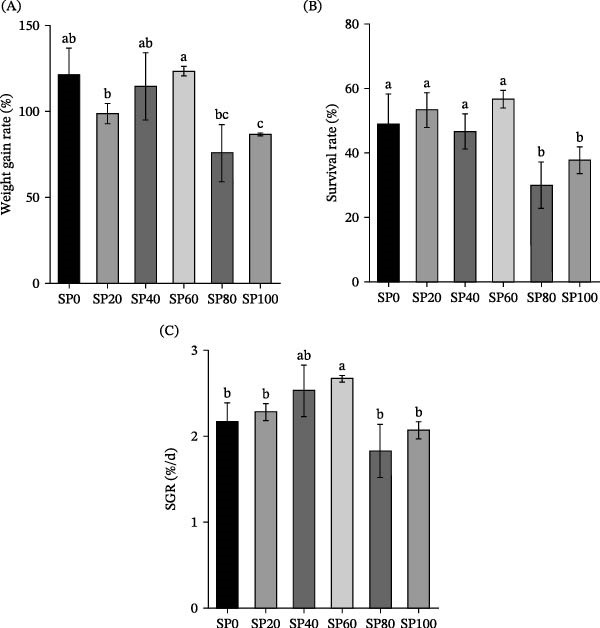
Weight gain rate, survival rate, and SGR of *M. nipponense* fed diets with varying levels of dietary *S. platensis*. (A) Weight gain rate; (B) survival rate; and (C) SGR. Values are shown as mean ± SE (*n* = 3). Significant differences are indicated by different letters above the bars (*p* < 0.05).

The weight gain rate in the SP20, SP40, SP60, and SP80 groups did not differ significantly from the control group (SP0). No significant difference was observed between the SP60 and SP40 groups. However, the SP60 group exhibited a significantly higher weight gain rate than the SP20, SP80, and SP100 groups (*p* < 0.05, Figure 1A). Survival rate was not significantly affected by *S. platensis* inclusion up to 60% (SP0‐SP60 groups; *p*  > 0.05, Figure 1B). In contrast, both metrics sharply decreased in the SP80 and SP100 groups (*p* < 0.05). The SP60 group showed significantly higher SGR than the SP0, SP20, SP80, and SP100 groups (*p* < 0.05), while the SP40 group did not differ significantly from those groups. Among all treatments, the SP60 group consistently exhibited the highest numerical values for growth, survival, and SGR.

### 3.2. Digestive Enzymes

**Figure 2:**
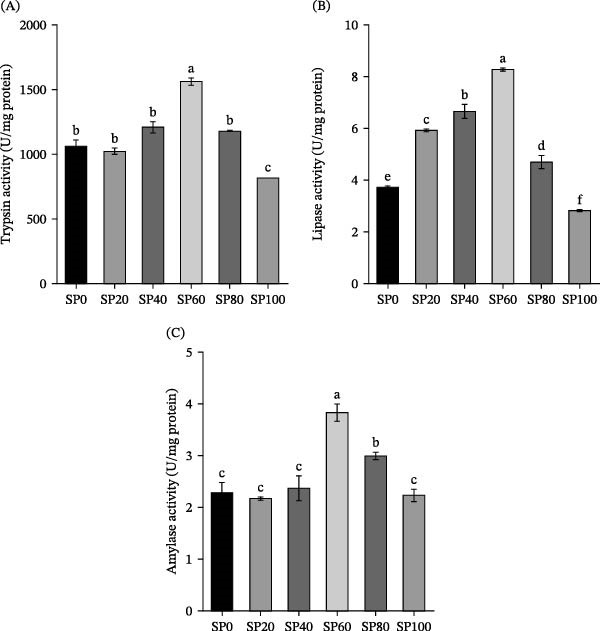
Digestive enzyme activities in *M. nipponense* fed diets with varying levels of *S. platensis*. (A) Trypsin activity; (B) lipase activity; (C) and amylase activity. Values are shown as mean ± SE (*n* = 3). Significant differences are indicated by different letters above the bars (*p* < 0.05).

The activities of key digestive enzymes in the hepatopancreas responded distinctly to dietary *S. platensis* levels (Figure 2). The SP60 group consistently exhibited the highest activities for all three enzymes. Specifically, its trypsin activity was significantly superior to all other groups (*p* < 0.05). While lipase activity was elevated in the SP20 to SP80 groups compared to the control (SP0), it peaked in the SP60 group and was lowest in SP100 (*p* < 0.05). Similarly, amylase activity was highest in the SP60 group, which was significantly greater than even the SP80 group, and both were higher than the remaining groups (*p* < 0.05).

### 3.3. Hepatopancreatic CAT and SOD Activities

**Figure 3:**
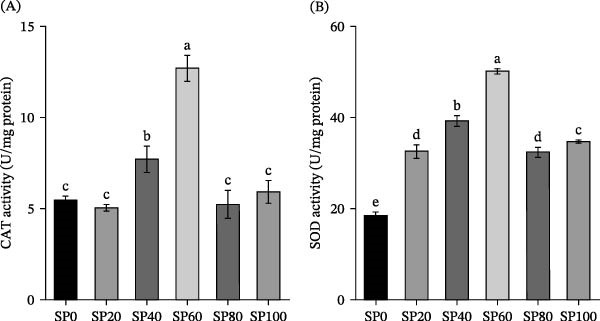
CAT and SOD activities in the hepatopancreas of *M. nipponense* fed diets with varying levels of *S. platensis*. (A) CAT activity and (B) SOD activity. Values are shown as mean ± SE (*n* = 3). Significant differences are indicated by different letters above the bars (*p* < 0.05).

CAT and SOD activities of the hepatopancreas were significantly modulated by dietary *S. platensis* (Figure 3). Both enzymes exhibited their peak activities in the SP60 group. Specifically, CAT activity was significantly elevated in the SP40 and SP60 groups compared to all other groups (*p* < 0.05), with the SP60 group being the highest. SOD activity demonstrated a generally increasing trend with higher *S. platensis* inclusion up to 60% (*p* < 0.05), and all experimental groups showed significantly greater activity than the SP0 control (*p* < 0.05).

### 3.4. Ovarian Development

#### 3.4.1. Validation of the Visual Staging Method by GSI

To confirm the reliability of the color‐based ovarian staging method under our experimental conditions, the GSI was measured for five representative females from each developmental stage (Stages I–V). As shown in Table 2, GSI values increased progressively with the advancing stage. Stage I prawns had the lowest GSI (mean ± SD), while Stage V prawn exhibited the highest GSI. These results validate the visual staging method, confirming that the color‐based classification accurately reflects quantitative changes in ovarian size associated with maturation.

**Table 2:** Gonadosomatic index (GSI) of each ovarian developmental stage in *M. nipponense*.

Developmental stage	Ovary color	Body weight (g)	Ovary weight (g)	GSI (%)
Ⅰ	Colorless transparency	0.7169 ± 0.22	0.0038 ± 0.0011	0.57 ± 0.22^a^
Ⅱ	White	0.8377 ± 0.23	0.0086 ± 0.0033	1.00 ± 0.15^a^
Ⅲ	Khaki	0.9633 ± 0.24	0.0133 ± 0.0038	1.38 ± 0.20^a^
Ⅳ	Green	1.1357 ± 0.23	0.0314 ± 0.0110	2.79 ± 0.94^b^
Ⅴ	Dark green	1.8218 ± 0.30	0.1342 ± 0.0490	7.31 ± 2.10^c^

*Note:* Data are presented as mean ± SE (*n* = 5). Different superscript letters indicate significant differences (*p* < 0.05) among different ovarian developmental stages.

#### 3.4.2. Effects of Dietary Fishmeal Replacement on Ovarian Maturation Rate

**Figure 4:**
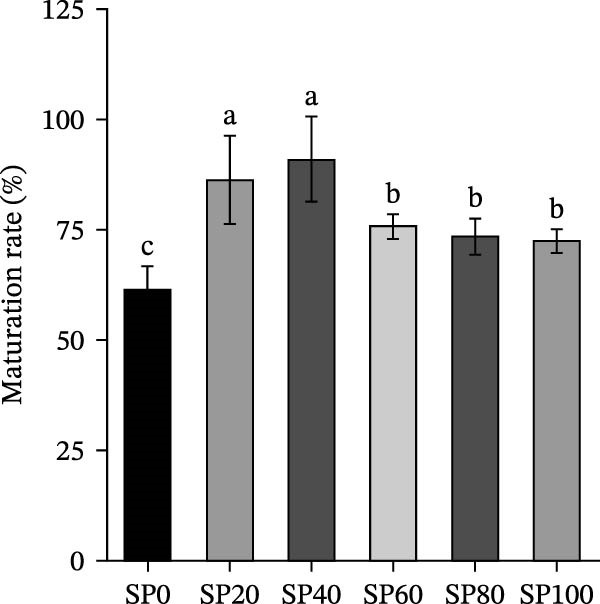
Ovarian maturation rate of *M. nipponense* fed diets with varying levels of *S. platensis*. Values are shown as mean ± SE (*n* = 3). Significant differences are indicated by different letters above the bars (*p* < 0.05).

The ovarian maturation rate was significantly influenced by dietary *S. platensis* inclusion (Figure 4). All replacement groups (SP20 to SP100) exhibited significantly higher maturation rates than the SP0 control group (*p* < 0.05). The most pronounced effects were observed in the SP20 and SP40 groups, with both groups exhibiting significantly higher values than those of the other treatments.

### 3.5. Amino Acid Analysis

Amino acid analysis of muscle tissue revealed that fishmeal substitution with *S. platensis* specifically altered the profile of certain amino acids without affecting the overall nutritional quality in terms of total (TAA) or umami amino acids (UAA), which remained stable across all groups (*p* > 0.05, Table 3). However, significant and dose‐dependent alterations were detected in several individual amino acids. Most notably, the EEA arginine decreased significantly in the SP60, SP80, and SP100 groups compared to the lower substitution groups (*p* < 0.05), while proline exhibited a converse trend, increasing significantly in these high‐substitution groups (*p* < 0.05). Regarding total EAAs, a significant reduction was observed in the SP60 and SP100 groups compared to the SP0 control (*p* < 0.05). Furthermore, in the SP100 group, five specific EAAs—arginine, threonine, methionine, leucine, and lysine‐were significantly decreased compared to SP0 (*p* < 0.05).

**Table 3:** Muscle amino acid composition (g/100 g) of *M. nipponense* fed diets with varying levels of *S. platensis*.

Amino acids	SP0	SP20	SP40	SP60	SP80	SP100
Essential amino acids (EAA)						
Histidine	0.45 ± 0.02	0.41 ± 0.01	0.44 ± 0.01	0.40 ± 0.00	0.43 ± 0.02	0.42 ± 0.01
Arginine	1.72 ± 0.03^a^	1.72 ± 0.05^a^	1.72 ± 0.04^a^	1.61 ± 0.02^b^	1.60 ± 0.05^b^	1.62 ± 0.06^b^
Threonine	0.86 ± 0.03^a^	0.82 ± 0.03^a^	0.82 ± 0.02^ab^	0.79 ± 0.02^ab^	0.83 ± 0.01^a^	0.80 ± 0.00^b^
Valine	0.87 ± 0.02	0.82 ± 0.01	0.86 ± 0.01	0.83 ± 0.02	0.88 ± 0.02	0.86 ± 0.01
Methionine	0.60 ± 0.01^a^	0.56 ± 0.02^ab^	0.58 ± 0.01^a^	0.55 ± 0.02^ab^	0.56 ± 0.01^ab^	0.54 ± 0.01^b^
Phenylalanine	0.80 ± 0.03	0.76 ± 0.02	0.79 ± 0.01	0.78 ± 0.02	0.82 ± 0.01	0.78 ± 0.00
Isoleucine	0.87 ± 0.02	0.82 ± 0.01	0.85 ± 0.01	0.83 ± 0.02	0.87 ± 0.01	0.85 ± 0.00
Leucine	1.54 ± 0.03^a^	1.45 ± 0.03^b^	1.49 ± 0.02^ab^	1.45 ± 0.04^ab^	1.51 ± 0.01^ab^	1.46 ± 0.01^b^
Lysine	1.73 ± 0.03^a^	1.61 ± 0.10^ab^	1.72 ± 0.06^ab^	1.62 ± 0.07^ab^	1.63 ± 0.07^ab^	1.52 ± 0.04^b^
Total EAA	9.45 ± 0.16^a^	8.97 ± 0.25^ab^	9.25 ± 0.14^ab^	8.86 ± 0.18^b^	9.13 ± 0.17^ab^	8.85 ± 0.74^b^
Nonessential amino acids (NEAA)						
Aspartic acid ^∗^	2.27 ± 0.06	2.16 ± 0.07	2.20 ± 0.01	2.20 ± 0.06	2.25 ± 0.03	2.23 ± 0.00
Glutamic acid ^∗^	3.51 ± 0.06	3.43 ± 0.12	3.43 ± 0.05	3.37 ± 0.08	3.45 ± 0.04	3.42 ± 0.02
Glycine ^∗^	1.44 ± 0.05	1.36 ± 0.05	1.40 ± 0.07	1.48 ± 0.06	1.51 ± 0.06	1.40 ± 0.04
Alanine ^∗^	1.18 ± 0.03	1.12 ± 0.04	1.15 ± 0.03	1.12 ± 0.04	1.17 ± 0.03	1.12 ± 0.01
Serine	0.89 ± 0.02	0.79 ± 0.02	0.82 ± 0.01	0.77 ± 0.02	0.83 ± 0.01	0.79 ± 0.01
Tyrosine	0.64 ± 0.02	0.60 ± 0.01	0.63 ± 0.01	0.61 ± 0.02	0.63 ± 0.01	0.61 ± 0.00
Cystine	0.08 ± 0.00	0.07 ± 0.00	0.07 ± 0.00	0.07 ± 0.01	0.07 ± 0.00	0.07 ± 0.00
Proline	0.45 ± 0.01^c^	0.53 ± 0.02^b^	0.52 ± 0.04^b^	0.62 ± 0.02^a^	0.62 ± 0.11^a^	0.60 ± 0.05^a^
Total NEAA	10.46 ± 0.40	10.06 ± 0.44	10.21 ± 0.29	10.24 ± 0.49	10.53 ± 0.30	10.24 ± 0.03
Total UAA	8.41 ± 0.34	8.07 ± 0.41	8.18 ± 0.21	8.17 ± 0.40	8.38 ± 0.11	8.17 ± 0.12
TAA	19.92 ± 0.39	19.03 ± 0.50	19.47 ± 0.26	19.10 ± 0.46	19.66 ± 0.33	19.10 ± 0.08

*Note:* Amino acids marked with an asterisk ( ^∗^) are umami amino acids. Data are presented as mean ± SE (*n* = 3). Significant differences (*p* < 0.05) among dietary groups for each amino acid are indicated by different superscript letters.

Abbreviations: EAA, essential amino acid; NEAA, nonessential amino acid; TAA, total amino acid; UAA, umami amino acid.

## 4. Discussion

### 4.1. Hepatopancreatic Digestive Profile

Digestive enzyme activity serves as a critical indicator of nutrient utilization capacity and physiological adaptation to dietary changes in aquatic species [35–37]. In the present study, all three key digestive enzymes (trypsin, lipase, and amylase) exhibited a consistent nonlinear response to spirulina substitution, peaking at the 60% replacement level (SP60). This coordinated enhancement suggests an optimal stimulation of the prawns’ overall digestive capacity at this substitution rate, which aligns with the superior growth performance observed in the SP60 group. Similar digestive improvements with moderate spirulina inclusion have been reported in *Litopenaeus vannamei* [38], *Ctenopharyngodon idella* [39], and *Oreochromis niloticus* [40], supporting the broad applicability of microalgae in enhancing the digestive function. This elevation in digestive enzyme activities is attributed to the presence of spirulina cell wall polysaccharides [41], which render the algae difficult to digest, thereby necessitating increased digestive enzyme production as an adaptive response in the prawn.

In contrast, the SP100 group showed a significant decline in trypsin and lipase activities compared to those of all other treatments. This reduction is likely multifactorial, with high spirulina inclusion potentially compromising feed palatability [42] and leading to reduced feed intake—an observation corroborated by the substantial residual feed noted in the SP100 tanks during the trial. The consequent nutritional deficiency presumably contributed to the suppressed enzyme synthesis and the poorest growth performance in this group.

Notably, lipase activity was significantly elevated in all spirulina‐fed groups except SP100 compared to the fishmeal‐based control. This specific increase may reflect spirulina‐induced modulations in lipid metabolism, possibly through the provision of beneficial fatty acids or the activation of lipid utilization pathways. The abundant polyunsaturated fatty acids (PUFAs) in *S. platensis*, particularly linoleic acid and γ‐linolenic acid [43], may contribute to enhanced lipid digestibility by serving as natural substrates that upregulate lipase expression or activity. Furthermore, microalgae‐derived fatty acids have been shown to increase docosahexaenoic acid (DHA) deposition in fish tissues [44, 45], suggesting that spirulina may improve lipid assimilation by providing a more favorable fatty acid profile for digestive enzyme recognition and absorption [46]. Similar lipid metabolic responses to spirulina supplementation have been documented in *Megalobrama amblycephala* [46] and *Danio rerio* [47], further supporting its role in regulating lipid digestive physiology.

### 4.2. Hepatic SOD and CAT Activities

The current study revealed that SOD activity was elevated in all spirulina‐fed groups, while CAT activity was significantly increased only in the SP40 and SP60 groups. This finding aligns with reports in other aquatic species, where spirulina inclusion boosted plasma SOD activity in gibel carp (*Carassius auratus gibelio*) [48] and upregulated the gene expression of both SOD and CAT in rainbow trout (*Oncorhynchus mykiss*) [49].

Phycocyanin, a prominent phycobiliprotein pigment extracted from the cyanobacterium *S. platensis*, is well recognized for its potent antioxidant properties [50, 51]. It enhances endogenous antioxidant defenses by increasing the activity of enzymes, such as SOD and CAT. These antioxidant enhancements are likely attributable to bioactive compounds in spirulina—particularly phycocyanin and its chromophore phycocyanobilin—which directly scavenge free radicals, enhance CAT activity, and modulate p38 MAPK and NF‐κB signaling pathways [52], thereby stimulating the body’s innate free radical scavenging systems [53, 54].

We acknowledge that a more comprehensive set of antioxidant biomarkers (e.g., GSH/GSSG ratio, MDA, GSH‐Px, ROS, and T‐AOC) would strengthen the conclusions. Due to sample limitations, these were not measured in the present study. Nevertheless, the significant and consistent elevation of both SOD and CAT, key primary antioxidant enzymes, in the SP60 group provides robust evidence for improved antioxidant status. Future studies should include a broader panel of oxidative stress markers.

### 4.3. Reproductive Performance

The significantly higher ovarian maturation rates observed in all spirulina‐fed groups demonstrate that *S. platensis* supplementation actively promotes ovarian development in *M. nipponense*. This finding is consistent with its role in enhancing oocyte formation in other aquatic species [55]. This effect is likely facilitated by key bioactive compounds in spirulina, such as phycocyanin and β‐carotene, which are known to preserve oocyte integrity and enhance developmental competence [56]. The underlying mechanisms may involve high protein and high‐quality amino acids in spirulina providing sufficient raw materials for the synthesis of vitellogenin [55, 57], along with the antioxidant protection of oocytes by phycocyanin [52]. The present study assessed ovarian development using the noninvasive visual staging method, which is well‐established for *M. nipponense* [26, 27]. However, we acknowledge that additional metrics—such as fecundity, egg diameter, and egg amino acid and fatty acid profiles—would provide a more comprehensive evaluation of reproductive performance. Future studies with extended experimental timelines that include spawning and egg collection are needed to fully elucidate the effects of *S. platensis* on reproduction.

Importantly, as noted in the introduction, *M. nipponense* is prone to precocious maturation under certain conditions, a phenomenon characterized by reduced somatic growth due to early energy allocation to ovarian development. Therefore, the practical value of enhanced ovarian development depends critically on whether growth performance is maintained. In the present study, spirulina substitution levels ≤60% did not impair growth compared to the control group. Thus, the observed promotion of ovarian development at these substitution levels does not represent undesirable precocious maturation but rather a beneficial effect that enhances reproductive potential without compromising somatic growth.

### 4.4. Muscular Amino Acids Profile

Substitution of fishmeal with spirulina did not significantly alter the TAA content or the levels of key UAAs in the muscle of *M. nipponense*, indicating a stable foundation for sensory quality. This stability in amino acid profiles has been consistently observed in other aquaculture species, including *M. amblycephala* [46] and *Ompok pabda* [11].

However, significant alterations were noted in the EAA metabolism. The SP100 group showed marked reductions in arginine, threonine, methionine, lysine, and leucine, aligning with the general decline in the EAA content reported in *O. pabda* at high substitution levels [11]. The reduction in muscle methionine and lysine can be linked to their lower levels in the spirulina‐substituted feed [58, 59]. Notably, a different pattern was observed for histidine as its muscle content was maintained despite reduced dietary intake. This observation merits further investigation.

A particularly noteworthy finding was that the SP60 group, which exhibited optimal weight gain, displayed significantly lower muscle levels of arginine and lysine compared to the control, despite all spirulina‐supplemented groups showing higher dietary arginine and lower lysine availability than the control group. This dissociation between muscle amino acid deposition and dietary input suggests that postabsorptive amino acid metabolism is influenced by factors beyond mere dietary concentration.

This phenomenon finds a parallel in *M. amblycephala*, where the diet yielding the best growth performance (3% spirulina supplementation) resulted in lower muscle levels of four EAAs compared to the control group [46]. The simultaneous enhancement of growth and reduction in EAA deposition suggest a physiological state of high metabolic efficiency and optimized nutrient utilization rather than a simple nutritional deficiency. These consistent cross‐species observations demonstrate that amino acid utilization is governed not merely by their dietary supply but, more critically, by the overall balance and bioavailability of the dietary amino acid profile. This principle is particularly relevant given the known deficiencies of *S. platensis* in histidine, lysine, and methionine relative to those in fishmeal [58, 59]. Such inherent imbalances likely induce species‐specific regulatory responses in metabolic pathways, which appear to prioritize sustaining overall growth and metabolic homeostasis over maximizing the deposition of certain amino acids in muscle tissues. This prioritization may be achieved through differential expression of amino acid transporters (e.g., SLC7 family members) [60] and differential utilization of amino acids for energy metabolism [61]. The precise molecular mechanisms driving this adaptive metabolic strategy remain to be fully elucidated and warrant further investigation.

Crystalline amino acids (histidine, lysine, and methionine) can be added to balance the deficiencies of *S. platensis* relative to fishmeal [58]. It would, therefore, be valuable for future studies to optimize the SP60 diet, for example, by supplementing limiting amino acids (particularly lysine and methionine) or using other nutritional strategies to further improve growth performance and potentially achieve even higher effective substitution levels.

### 4.5. Growth and Survival Performance

This study demonstrates that dietary fishmeal can be successfully replaced by *S. platensis* at inclusion levels up to 60% without incurring significant detrimental effects on the survival of *M. nipponense*. Among all treatments, the SP60 group exhibited the highest weight gain rate and SGR. Its weight gain rate was comparable to the SP40 and control groups but significantly higher than the SP20, SP80, and SP100 groups; its SGR was comparable to that of SP40 but significantly higher than that of all other groups. Therefore, considering growth performance and survival, the 60% replacement level (SP60) was identified as the optimal substitution level, supporting growth and survival comparable to those of the control and SP40 groups while outperforming higher replacement levels. This optimal substitution level is somewhat higher than the thresholds reported for many other aquatic species, including gibel carp (*C. auratus gibelio*) (50%) [48], Pabda catfish (*O. pabda*) (15%) [11], and juvenile grass carp (*C. Idella*) (5%) [39], highlighting a distinct species‐specificity in the utilization of this microalgal protein source. The successful substitution up to this level can be attributed to the robust nutritional composition of *S. platensis*, which provides a balanced array of EAAs, lipids, and micronutrients.

Despite the potential survivor bias associated with the high‐mortality groups, the poor performance of the SP80 and SP100 groups can be attributed to multiple synergistic factors: severe deficiencies in lysine, methionine, and histidine; reduced palatability evidenced by substantial residual feed [58, 59]; and a potential digestive burden imposed by spirulina cell wall polysaccharides on the short digestive tract of *M. nipponense* [41]. These factors collectively explain the significantly lower survival and growth in these groups and are consistent with the parallel declines in digestive and antioxidant enzyme activities reported above. Thus, while caution is warranted when interpreting data from these high‐mortality groups, the convergence of multiple lines of physiological evidence supports the identification of 60% as the optimal substitution threshold.

## 5. Limitations of the Study

Several limitations of this study should be acknowledged. First, the survival rates in the SP80 and SP100 groups fell below 30%, which may introduce a survivor bias. Consequently, the data from these groups should be interpreted with caution as they reflect only the surviving subset of animals. Nevertheless, the consistent decline in growth and survival across the SP80 and SP100 groups strongly suggests that substitution levels of 80% and 100% are unsuitable for *M. nipponense* under the present formulation.

Second, all experimental diets contained 17% α‐cellulose as a pellet‐binding agent. This high level of indigestible fiber was necessary because high inclusion levels of *S. platensis* (particularly in the SP100 diet) impaired pelletability, and preliminary trials showed that conventional cellulose alone could not produce stable pellets at these high substitution levels. Although α‐cellulose is often considered an inert filler [62], such a high level of indigestible fiber may influence nutrient digestibility, gastrointestinal transit time, and feed intake. However, because the same level of α‐cellulose was used consistently across all treatment groups, the comparative conclusions—specifically, that 40%–60% substitution yields the best overall performance—remain valid. Importantly, the primary goal of this study was to identify the optimal substitution level under standardized conditions, not to formulate a commercially ready diet. Future studies should explore reducing α‐cellulose to the minimum required for pelletability or testing alternative binders (e.g., gelatin and alginate) to better approximate practical aquafeed formulations.

We also note that the primary objective of this study was to evaluate the effects of spirulina substitution on ovarian development and associated physiological responses, with growth parameters included as supporting indicators. Future studies with extended trial durations (e.g., 60–90 days) are needed to fully assess the long‐term growth performance, true feed efficiency, and muscle nutrient deposition.

## 6. Conclusions

In conclusion, substitution of up to 60% of fishmeal with *S. platensis* is an optimal strategy for *M. nipponense*. This dietary strategy significantly enhanced digestive functions and increased the activities of SOD and CAT while promoting ovarian development without compromising growth performance or survival rate. From an applied perspective, given that the SP60 diet performed well without amino acid supplementation, future research should explore supplementing the limiting amino acids (particularly lysine and methionine) and reducing the α‐cellulose content to the minimum required for pelletability in order to further improve performance and potentially enable even higher effective substitution levels.

## Author Contributions

**Weihong Zhao**: conceptualization, methodology, funding acquisition, supervision, writing – review and editing. **Longyu Liu**: formal analysis, writing – original draft, writing – review and editing. **Anran Zou**, **Fengjuan Jiang**, **Da Yue**, and **Ling Yin**: investigation, resources. **Tianqi Cao**: data curation, writing – original draft, software. **Wenjing Wu**: investigation. **Chuanjie Qin** and **Zhigang Gao**: resources.

## Funding

This work was supported by the National Natural Science Foundation of China (Grant 31101887), the Jiangsu Provincial Natural Science Foundation (Grant BK2011419), the Key Research and Development Program (Industrial) of Yancheng City (Project Number BE2023026), and the Dongtai Cibainian Bioengineering Co., Ltd.

## Disclosure

All authors have reviewed the manuscript.

## Ethics Statement

The experimental animal used in this study was the freshwater prawn *Macrobrachium nipponense*, an invertebrate species; therefore, ethical approval from an animal care and use committee was not required.

## Conflicts of Interest

The authors declare no conflicts of interest.

## Data Availability

Data will be made available upon request.
